# Correlation between ADC, ADC ratio, and Gleason Grade group in prostate cancer patients undergoing radical prostatectomy: Retrospective multicenter study with different MRI scanners

**DOI:** 10.3389/fonc.2023.1079040

**Published:** 2023-02-20

**Authors:** Johan Bengtsson, Erik Thimansson, Erik Baubeta, Sophia Zackrisson, Pia Charlotte Sundgren, Anders Bjartell, Despina Flondell-Sité

**Affiliations:** ^1^Department of Clinical Sciences, Radiology, Lund, Lund University, Lund, Sweden; ^2^Department of Medical Imaging and Physiology, Skåne University Hospital, Lund, Sweden; ^3^Department of Translational Medicine, Lund University, Malmö, Sweden; ^4^Department of Radiology, Helsingborg Hospital, Helsingborg, Sweden; ^5^Department of Medical Imaging and Physiology, Skåne University Hospital, Malmö, Sweden; ^6^Lund Bioimaging Center (LBIC), Lund University, Lund, Sweden; ^7^Department of Urology, Skåne University Hospital, Malmö, Sweden

**Keywords:** MRI, MR-diffusion, ADC, neoplasms, prostate

## Abstract

**Background:**

MRI is an important tool in the prostate cancer work-up, with special emphasis on the ADC sequence. This study aimed to investigate the correlation between ADC and ADC ratio compared to tumor aggressiveness determined by a histopathological examination after radical prostatectomy.

**Methods:**

Ninety-eight patients with prostate cancer underwent MRI at five different hospitals prior to radical prostatectomy. Images were retrospectively analyzed individually by two radiologists. The ADC of the index lesion and reference tissues (contralateral normal prostatic, normal peripheral zone, and urine) was recorded. Absolute ADC and different ADC ratios were compared to tumor aggressivity according to the ISUP Gleason Grade Groups extracted from the pathology report using Spearman’s rank correlation coefficient (ρ). ROC curves were used to evaluate the ability to discriminate between ISUP 1-2 and ISUP 3-5 and intra class correlation and Bland-Altman plots for interrater reliability.

**Results:**

All patients had prostate cancer classified as ISUP grade ≥ 2. No correlation was found between ADC and ISUP grade. We found no benefit of using the ADC ratio over absolute ADC. The AUC for all metrics was close to 0.5, and no threshold could be extracted for prediction of tumor aggressivity. The interrater reliability was substantial to almost perfect for all variables analyzed.

**Conclusions:**

ADC and ADC ratio did not correlate with tumor aggressiveness defined by ISUP grade in this multicenter MRI study. The result of this study is opposite to previous research in the field.

## Introduction

Prostate cancer (PCa) is the most common cancer in men worldwide (GLOBOCAN 2020) (1). However, most men with PCa have low-grade, indolent tumors. Therefore, discriminating between indolent and aggressive tumors is a diagnostic issue. With the traditional diagnostic approach, which includes a blood test of prostate specific antigen (PSA), digital rectal examination, and systematic transrectal ultrasound-guided biopsies, only a small and randomly distributed fraction of the gland is examined, resulting in a substantial risk of both over- and under-sampling. A more modern pathway involves magnetic resonance imaging (MRI) to detect clinically significant prostate cancer (csPCa) and rule out other causes of elevated PSA levels. On pathology, csPCa is defined as a Gleason score ≥7 (including 3 + 4 with a prominent but not predominant Gleason 4 component), volume ≥0.5 mL, and/or extra prostatic extension (2). Today, the International Society of Urological Pathology (ISUP) grade is often used to categorize different Gleason score patterns (3). When using MRI as a triage tool, unnecessary biopsies can be avoided, and targeted when required. This approach was investigated in the PRECISION study, which showed that MRI followed by targeted biopsies detected more significant tumors (38% versus 26%, p=0.005) and fewer insignificant tumors (9% versus 22%, p<0.001) compared to systematic biopsies (4). In the group that had an MRI in the work up, 28% had a negative MRI and, thus, did not have to undergo biopsy. These results changed the work-up routine, and MRI is now a cornerstone of PCa diagnosis. Therefore, the demands on MRI are high in terms of technical quality and radiological interpretation for correctly detecting or excluding csPCa.

Prostate Imaging – Reporting and Data System (PI-RADS, version 2.1) is a system that describes how to perform, interpret, and report MRI of the prostate (2). The most important MRI sequence is diffusion-weighted imaging (DWI), which is the deciding sequence in the peripheral zone (PZ) and the secondary sequence in the transition zone (TZ). DWI provides information on tissue composition and tumor cellularity (5). The signal intensity on DWI reflects the motion of water molecules in the tissue. The concept is based on the theory that a tumor consists of more dense tissue than normal prostatic tissue.

Several studies have shown that the ADC value inversely correlates with ISUP grade and is often used as a marker of aggressiveness (5–9). Several cut-off values have been proposed; in PIRADS 2.0, a threshold of 750-900 µm^2^/s was suggested as a pathological ADC value, but no consensus has been reached (8, 10). The concept is associated with several difficulties. First, the ADC varies substantially depending on several factors, including the b-values used, scanner field strength, patient and coil geometry, temporal fluctuations in the magnet, and variations in measurements between readers. Furthermore, non-cancerous lesions, such as benign prostate hypertrophy, may also exhibit decreased ADC values, and there is a substantial overlap in ADC values and PCa (11). ADC is sometimes used as a marker of aggressiveness in other organs and diseases. For example, in rectal adenocarcinoma, a lower ADC value is associated with a more aggressive tumor and poorer survival rate. Similar correlations have been found in certain types of breast cancer, ovarian cancer, lung cancer, and gliomas (12–15).

A common way to overcome the differences in absolute ADC values is to normalize the ADC by using different ADC ratios (10, 16). The ADC ratio is expressed as the ratio between the ADC value of the tumor and the ADC value of another location, such as non-cancerous tissue in the same organ or other organs in the same patient (5, 17).

In recent years, several studies have investigated the potential benefit of using the ADC ratio over absolute ADC values. Some authors have affirmed that the ADC ratio is the preferred method and demonstrated significant capability in discriminating Gleason 3 + 4 from 4 + 3 PCa (5, 8, 9, 16, 18). Other authors have been more doubtful (19).

The aim of the present study was to investigate, in a consecutive patient cohort imaged using different MRI scanners, how absolute ADC value and ADC ratios correlate with ISUP grade following robot-assisted laparoscopic prostatectomy (RALP). A secondary aim was to assess the potential inter-observer variability.

## Material and methods

The study was a retrospective cohort study approved by the local ethics review committee at Lund University (Dnr 2014-886) and the Swedish ethical review authority (entry no. 2019-03674).

### Study population

**Figure 1:**
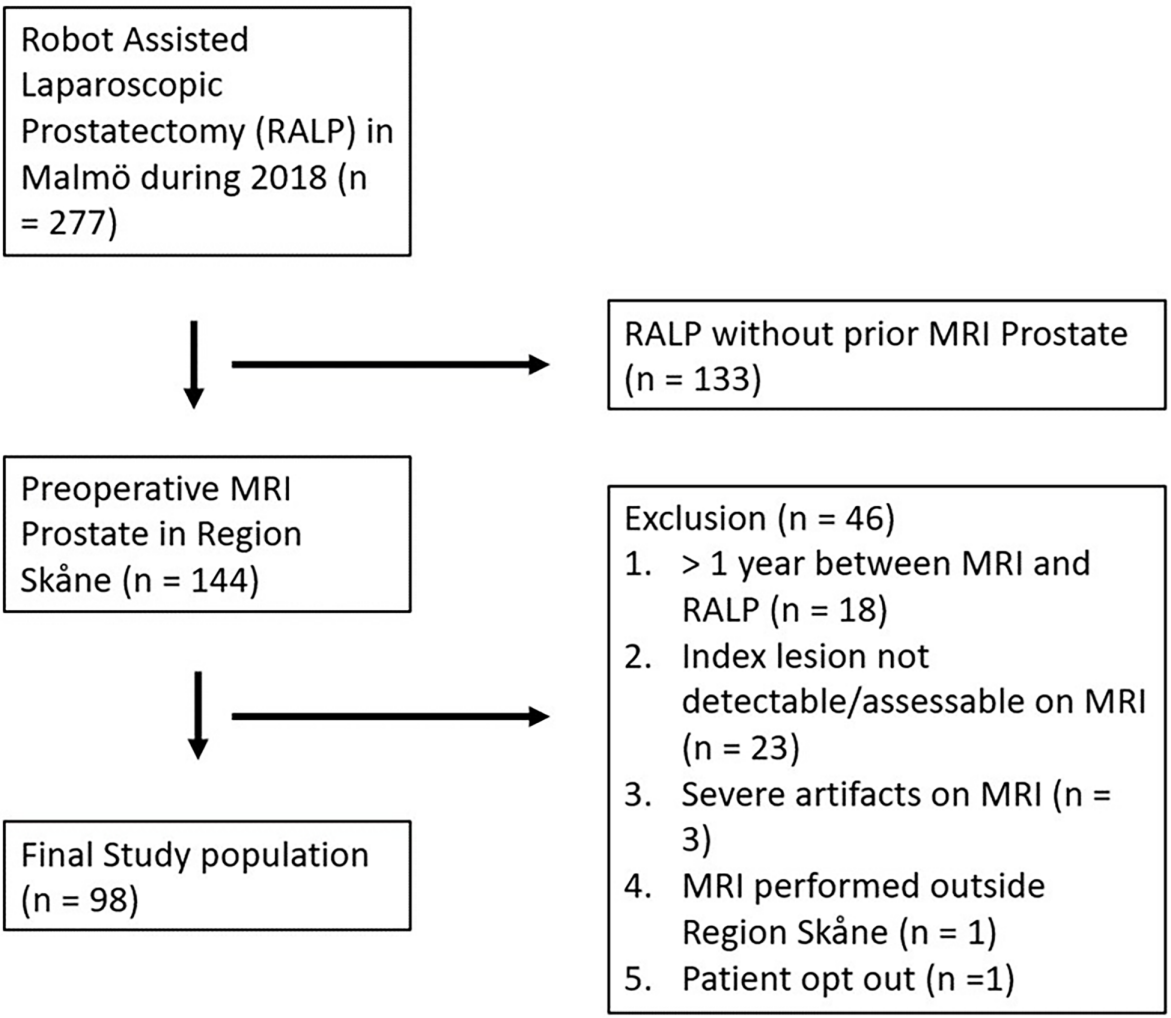
Flow diagram of patient selection.

All consecutive patients who underwent RALP for biopsy proven PCa at Skåne University Hospital in Malmö, Sweden, during 2018 were identified and assessed for eligibility. Patients were included if they had undergone MRI less than 1 year before surgery at five different hospitals. Patients were excluded if the index lesion described in the pathology report was not identified on MRI, severe artifacts were present on MRI, the MRI was performed outside of Region Skåne, or the patient opted out. Lesions were excluded based on consensus between two readers (JB and ET). The data collection algorithm is presented in **Figure 1**. Patient data were obtained from medical records.

### Pathological examination

The surgical specimens were handled according to clinical routines and fixed in formalin. Lesions were examined by experienced pathologists using hematoxylin and eosin staining. Pathological data and whole mount (WM) tumor maps were obtained from the pathology report. The location and Gleason score of the index lesion were recorded using the ISUP category classification (**Table 1**).

**Table 1:** ISUP grade groups and the corresponding Gleason scores and patterns.

ISUP grade group	Gleason score	Gleason pattern
1	≤6	≤3+3
2	7	3+4
3	7	4+3
4	8	4+4, 3 + 5, 5 + 3
5	9 or 10	4+5, 5 + 4, or 5 + 5

### MRI acquisition and image analysis

Preoperative MRI of the prostate was performed within Region Skåne using one of eight MRI scanners at five sites. Both 3T and 1.5T scanners were used. According to local routines, different imaging acquisition parameters were used at different sites. All protocols included transverse, coronal, and sagittal T2-weighted turbo spin-echo images, transverse T1-weighted images, diffusion-weighted images with a high b-value of 1500 s/mm^2^, and a calculated ADC map. A list of MRI scanners and imaging acquisition parameters for the DWI are presented in **Table 2**.

**Table 2:** Overview of scanners and diffusion-weighted imaging (DWI) acquisition parameters.

Scanner	Patients,n (%)	Vendor	Model	Field strength, T	Sequence	B-values*, s/mm^2^	TR, ms	TE, ms	Acquisition matrix	FOV,mm^2^	Slice thickness, mm	Interslice gap, mm	DWI acquisition time, min
1	36 (36.7)	Siemens	TrioTim	3	2D EPI	50, 400, 1500	5200	87	128	240 x 240	3	3.6	Not applicable
2	12 (12.2)	Siemens	Skyra	3	2D EPI	0, 800, 1500	6700	75	130	240 x 240	3	4	5:50
3	12 (12.2)	Siemens	Prisma	3	2D EPI	0, 800, 1500	4500	66	130	240 x 240	3	3	3:56
4	24 (24.5)	Siemens	Avanto fit	1.5	2D EPI	50, 300, 1000, 1500	3300	67	128	240 x 240	3	4	8:15 + 5:33**
5	7 (7.1)	Siemens	Avanto fit	1.5	2D EPI	50, 300, 1000	2900	62	128	240 x 240	3	3	5:33 + 2:37**
6	3 (3.1)	GE	Optima4550r	1.5	2D EPI	50, 400, 800	4746	72	256	280 x 280	4	4.5	3:52
7	2 (2.0)	Siemens	Aera	1.5	Resolve	50, 400, 800	4620	57	116	200 x 200	4	4	6:25 + 6:19**
8	2 (2.0)	Siemens	Avanto fit	1.5	Resolve	50, 400, 800	4620	58	116	200 x 200	4	4	6:41 + 4:41**

EPI, echo-planar imaging; TR, repetition time; TE, echo time; FOV, field of view. *B-values included in the ADC calculation, **separate b1500.

Two readers, both specialists in radiology with 4 and 5 years of experience in reading prostate MRI, performed all imaging analyses as described below. The examinations were reviewed using the clinical Picture Archiving and Communication System, Sectra IDS7.

**Figure 2:**
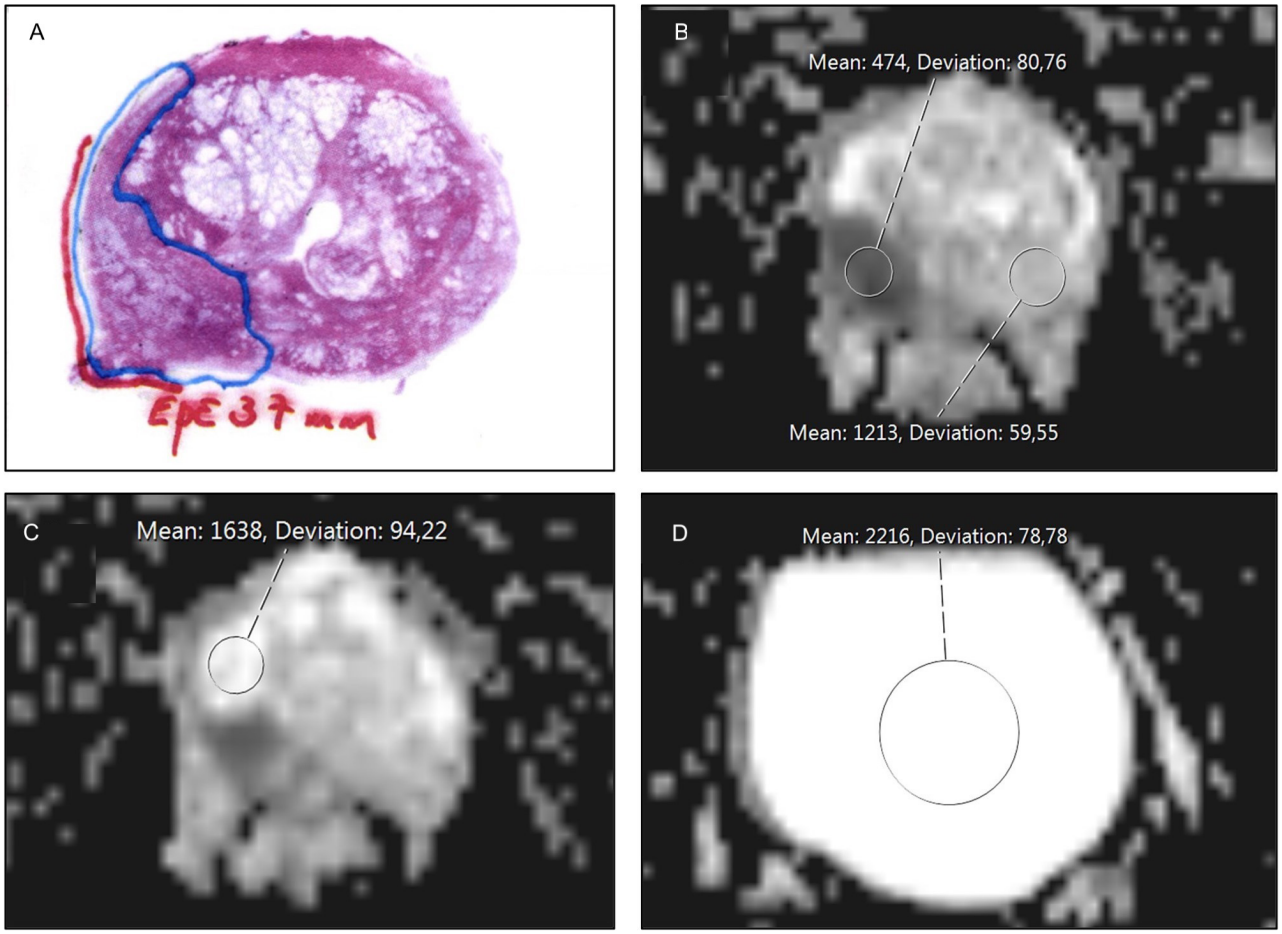
Example of a whole mount pathology specimen and placement of a region of interest (ROI) in the ADC map. The specimen was from a 70-year-old man with prostate cancer, PSA level 6.2 ng/mL, and clinical stage T3b. Systematic biopsies showed Gleason 4 + 5 (ISUP grade 5) in 7 of 12 cores. MRI was performed for staging and revealed a 2 x 3 cm PI-RADS 5 lesion in the right peripheral zone (PZ) with findings in line with extraprostatic extension (EPE) and seminal vesicle invasion (SVI). The final staging was pT3a. **(A)** Midgland whole mount specimen with a large tumor in the right PZ (blue border) with 37 mm EPE (red line). **(B)** Circular ROI in tumor (ADC_lesion_ = 474 x 10^-6^ mm^2^/s) and in contralateral non-tumorous tissue (ADC_contralat ref_ = 1213 x 10^-6^ mm^2^/s). **(C)** Circular ROI drawn in non-tumorous PZ (ADC_PZ ref_ = 1638 x 10^-6^ mm^2^/s). **(D)** Circular ROI in urinary bladder (ADC_urine ref_ = 2216 x 10^-6^ mm^2^/s). Tumor to non-tumor ratio = 0.36, tumor to PZ ratio = 0.29, and tumor to urinary bladder ratio = 0.21.

First, and in consensus, the two readers matched the index lesion in the surgical specimen with the corresponding lesion on MRI using the pathological report and the whole-mount tumor map. In a second step, the remaining interpretation and image analyses were performed individually. For the index lesion, each reader recorded the maximum diameter in millimeters, zone location (PZ or TZ), and PI-RADS score (version 2.1). A circular region of interest (ROI) was placed in the index lesion in the ADC map on the slice with the largest cross-sectional area of tumor (ADC_lesion_). The ROI was drawn to include only the lesion without any surrounding parenchyma. The size of the ROI was not fixed, it was drawn as big as possible within the defined lesion. A second ROI (ADC_contralat ref_) of the same size was placed at the contralateral position on the same slice, that is in the same zone as the index lesion. A third and fourth ROI was placed in the most homogenous area in the PZ (ADC_PZ ref_) and in the urinary bladder (ADC_urine ref_), respectively. For each ROI, the mean ADC value was recorded (**Figure 2**).

### Statistical analysis

Descriptive statistics were used to present the study population. Box plots and Spearman’s rank correlation coefficient (ρ) were used to evaluate the association between ISUP grade and ADC variables. Measurements from reader 1 were used for the analyses of ADC metrics. These analyses were repeated and stratified by scanner field strength (1.5 vs. 3T) and tumor location (PZ vs. TZ). Receiver operating characteristic (ROC) curves were used to evaluate the ability to discriminate between ISUP 1-2 and ISUP 3-5 based on ADC variables. Interrater reliability was evaluated using Bland-Altman plots and intraclass correlation (ICC) based on the formula for random effects, absolute agreement, and single rater measurements. The ICC values were rated as follows: slight agreement, 0 – 0.20; fair agreement, 0.21 – 0.40; moderate agreement, 0.41 – 0.60; substantial agreement, 0.61 – 0.80; almost perfect agreement, 0.81 – 1.

All statistical analyses were performed in R version 4.0.2. The pROC package was used for ROC curves and the irr package to calculate ICC.

## Results

A total of 144 men underwent RALP due to biopsy proven PCa and had an MRI prior to the procedure. After exclusion for different reasons (**Figure 1**), 98 patients were included in the final study analysis. The patient and tumor characteristics are presented in **Table 3**. No specimen was classified as ISUP 1. Most index lesions were located in the PZ of the prostate. Patients with different ISUP grades were relatively evenly distributed over the eight scanners, details are available in **Supplementary Table 1**.

**Table 3:** Patient characteristics (n=98).

Characteristic	*Mean ± SD (min – max)*
Age, years	66.3 ± 6.4 (45 – 76)
Time between MRI and RALP, months	4.08 ± 2.6 (1 – 11)
Preoperative PSA, ng/mL	9.26 ± 6.8 (1.8 – 39.0)
	*n (%)*
Clinical T-stage	
T0	4 (4.1)
T1	13 (13.3)
T1c	29 (29.6)
T2	36 (36.7)
T2b	4 (4.1)
T2c	2 (2.0)
T3	9 (9.2)
T3a	1 (1.0)
Pathological T-stage	
T1	0 (0)
T2	51 (52.0)
T3a	34 (34.7)
T3b	12 (12.2)
T3	0 (0)
Missing	1 (1.0)
Biopsy ISUP grade	
1	7 (7.1)
2	41 (41.8)
3	24 (24.5)
4	9 (9.2)
5	17 (17.3)
Pathological ISUP grade	
1	0 (0)
2	39 (39.8)
3	41 (41.8)
4	3 (3.1)
5	15 (15.3)
MRI field strength	
1.5 Tesla	38 (38.8)
3 Tesla	60 (60.1)
Zone location	
Peripheral zone	68 (69.4)
Transitional zone	30 (30.6)

### ADC measurements vs. ISUP grade

**Figure 3:**
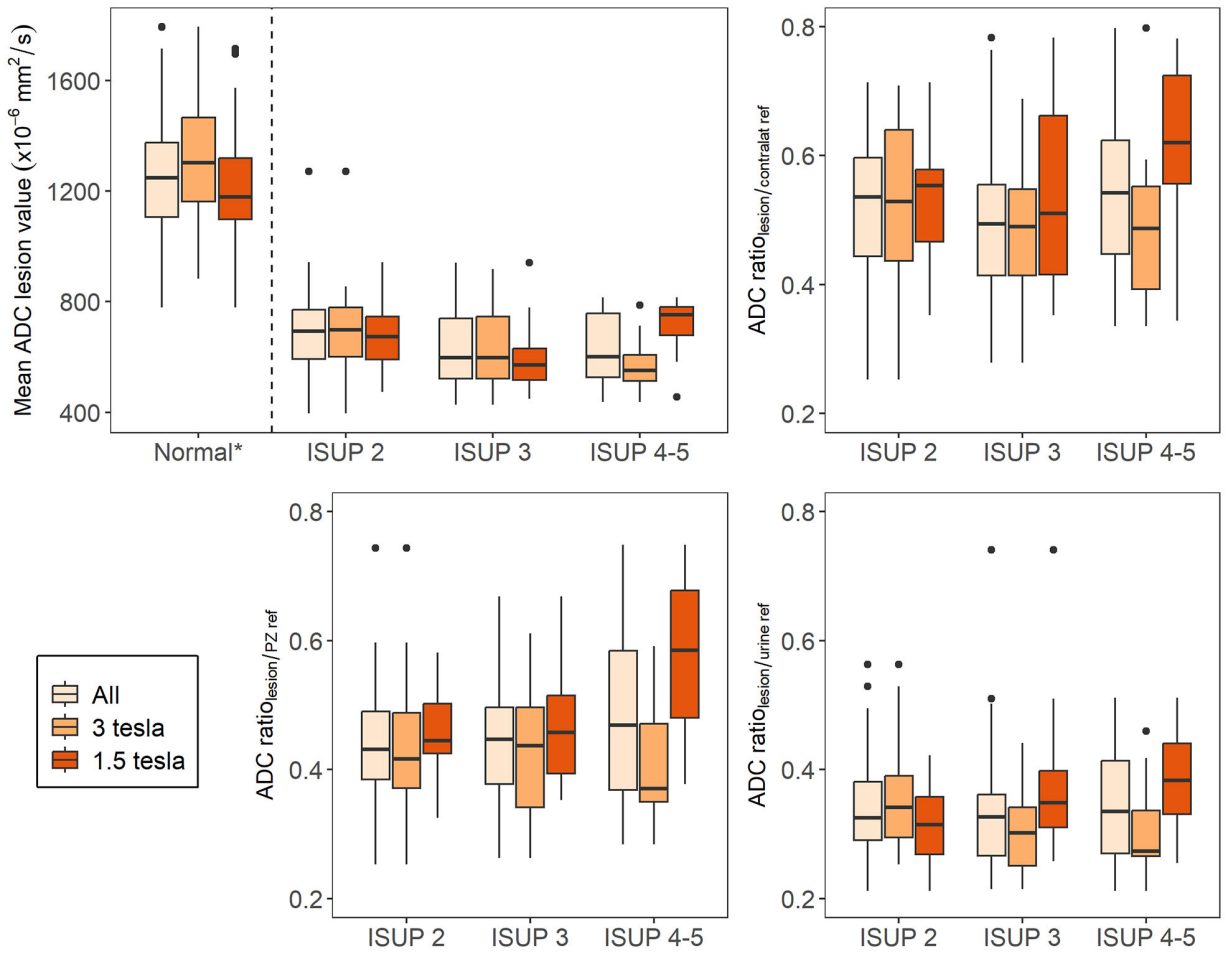
Box-and-whisker plots of apparent diffusion coefficient (ADC) metrics for tumors stratified by ISUP grade. (*) *Normal* represents the absolute ADC value of the normal appearing tissue in the contralateral position of the index lesion.

The average ADC_lesion_ was 652×10^-6^ mm^2^/s (range 396×10^-6^ mm^2^/s to 1271×10^-6^ mm^2^/s), whereas the average ADC_contralat ref_ tissue was 1275×10^-6^ mm^2^/s (range 779×10^-6^ mm^2^/s to 1794×10^-6^ mm^2^/s). The average ADC_PZ ref_ was 1478×10^-6^ mm^2^/s (range 779×10^-6^ mm^2^/s to 2155×10^-6^ mm^2^/s) and of ADC_urine ref_ was 2021×10^-6^ mm^2^/s (range 861×10^-6^ mm^2^/s to 3368×10^-6^ mm^2^/s; **Figure 3**). We found no significant negative correlation, between absolute the ADC value of the index lesion and the ISUP grade. The observed spearman correlation between the ADC of the index lesion and ISUP grade was low (ρ= -0.18) and not significant. Furthermore, the ADC of the index lesion did not perform well in discriminating between ISUP 1-2 and ISUP 3-5 (AUC= 0.62 [95% CI 0.51-0.74]). A tendency for a negative correlation was observed when the results from the 3T scanners were analyzed separately (ρ= -0.27; p<0.05), but not for the 1.5 T scanners (ρ= -0.01). Tables reporting the correlation values stratified by field strength are available in **Supplementary Table 2**. We found no correlation in separate analyses of the PZ and TZ.

**Figure 4:**
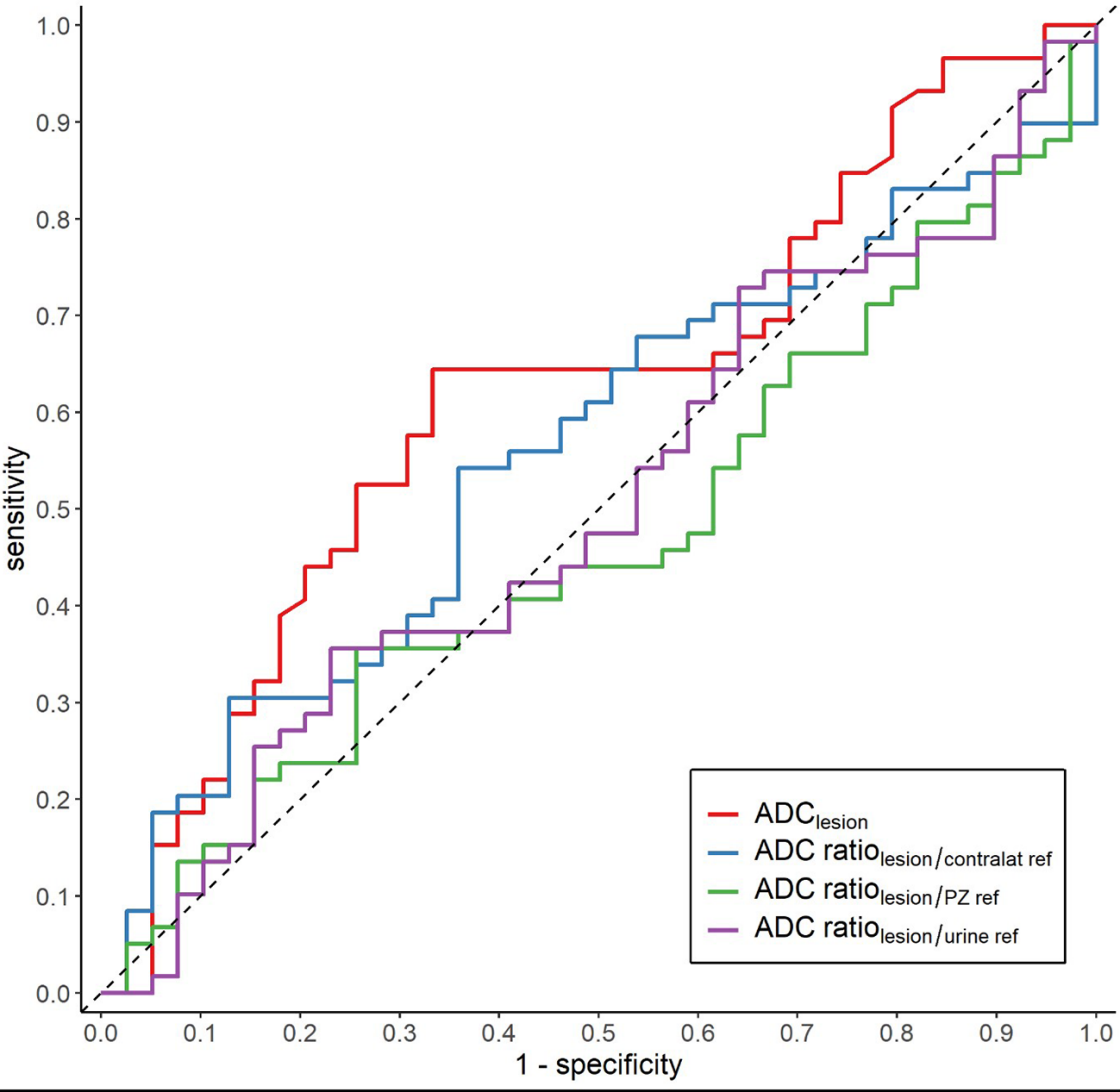
ROC curves comparing absolute ADC and three different ADC ratios in discriminating ISUP 1-2 from ISUP 3-5.

The three different ADC ratios were calculated for each lesion (ADC_lesion_/ADC_contralat ref_, ADC_lesion_/ADC_urine ref_, and ADC_lesion_/ADC_PZ ref_ in relation to tumor aggressiveness. None of them showed any discriminatory effect (**Figures 3**, **4**).

**Figure 5:**
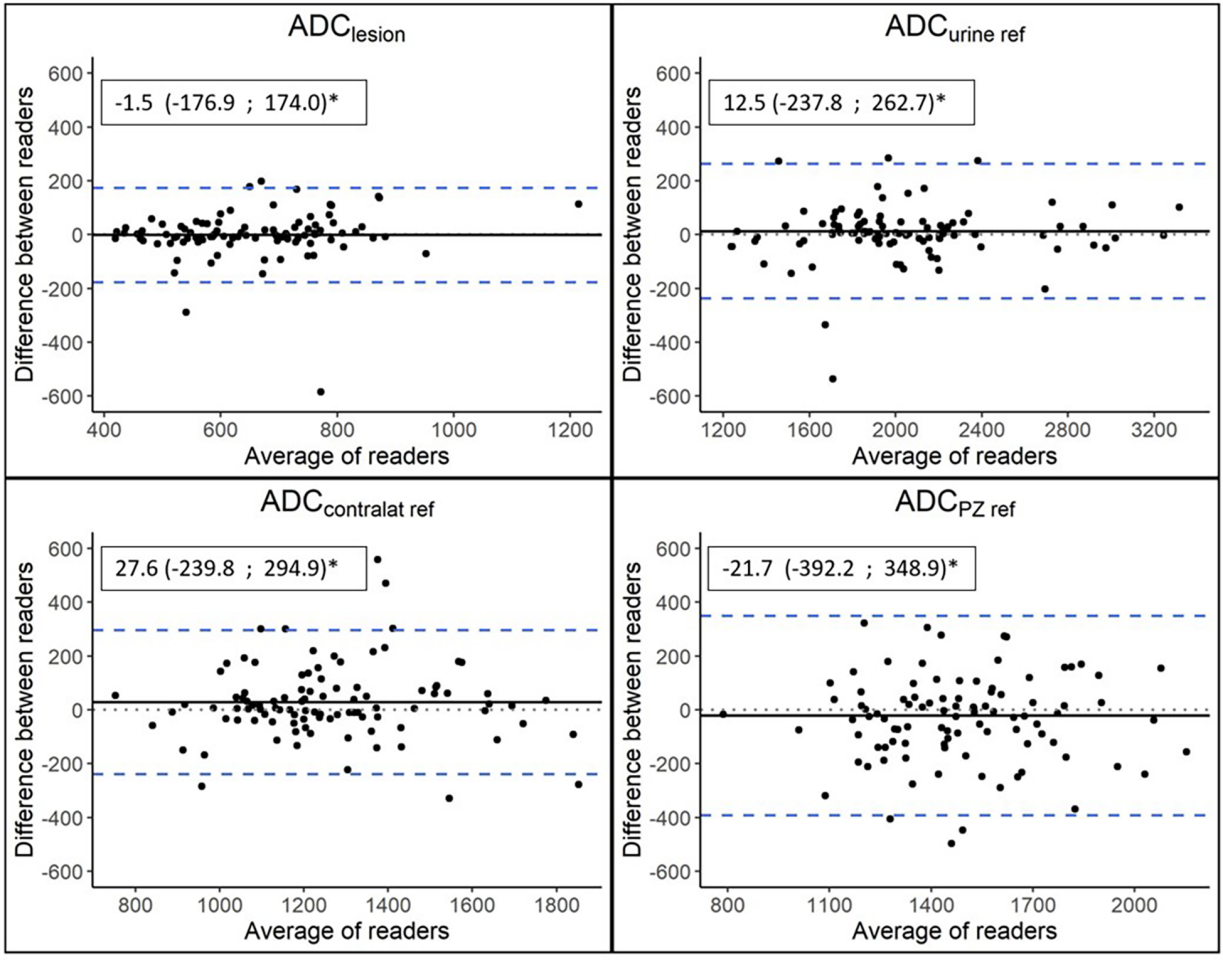
Bland-Altman plots. The dotted lines represent no difference between readers, the solid lines represent mean differences between readers, and the dashed blue lines represent limits of agreement, calculated as the mean difference ± 1.96SD of the mean difference. *Mean differences between readers (95% limits of agreement).

The agreement between the two readers in the ADC measurements was almost perfect for ADC_lesion_ (ICC of 0.80 [95% CI 0.72 – 0.86]), ADC_contralat ref_ (ICC of 0.82 [95% CI 0.75 – 0.88]), and ADC_urine ref_ (ICC of 0.96 [95% CI 0.94 – 0.97]). For ADC_PZ ref_, the agreement was substantial (ICC of 0.75 [95% CI 0.65 – 0.86], **Figure 5**).

## Discussion

This multi-scanner cohort study of 98 consecutive patients with MRI of the prostate before RALP showed no correlation between the absolute ADC value of the tumor and tumor aggressivity determined by pathology. No improvement was noted when the ADC value was normalized by applying different ADC ratios. Thus, no threshold values for ADC or ADC ratio were determined to discriminate significant from non-significant PCa. The inter-reader agreement between the two observers was substantial to almost perfect.

Different methods of interpretation have been applied to predict whether a lesion found on MRI represents benign tissue, non-significant cancer, or significant cancer. When comparing the results from the different studies, the definition of csPCa is crucial, as most authors try to define a threshold value for different ADC metrics in relation to tumor aggressivity. Some papers have used ISUP grade 1 as non-significant and ISUP 2 and higher as significant (20–24), whereas others have included ISUP 2 in the non-significant group. One study even included all ISUP 2 and 3 in the more harmless group and used the terms intermediate and high-risk cancer as the border between the two groups (25). Boesen et al. performed their analyses on two different cut-offs with ISUP 2 in both the significant and non-significant groups (8). In our study, all resected prostates were ISUP 2 or higher, which gave us no choice to use only ISUP 1 in the non-significant group. This was also true for the 23 patients in whom the index lesion could not be identified on MRI.

Regardless of which definition of csPCa is used, several authors have reported a strong inverse correlation between ADC metrics and tumor aggressivity, with a reported AUC of up to 0.94 (26) or 0.96 (17). This contrasts with the results of our study, as we found an AUC of 0.62, which would suggest that the absolute ADC value is not useful for predicting the presence of csPCa. The reasons for these results can be debated. We used eight different MRI scanners with different acquisition parameters. Disparate absolute ADC values are not unexpected with these settings. Barret et al. calculated different ADC values from the same scans by combining four b-values in different ways, thereby simulating different parameters (5). Most combinations showed a relatively good inverse correlation with tumor aggressivity. When they used the ratio between tumorous and non-tumorous ADC values, the differences in acquisition parameters were less obvious. Thus, they stated that the ADC ratio may be considered a more robust tool for assessing restricted diffusion in the prostate (5). With the same intention, we evaluated whether the disparate ADC values between our scanners could be more useful when different ratios were applied. However, despite using three different tissues as denominators in the creation of the ratios, no added value or better performance were found for the metrics. In fact, the AUC was even smaller, close to 0.5 for all three ratios, which is slightly smaller than for the absolute ADC. For the 1.5T scanners there was a tendency of positive correlation, instead of the expected negative correlation, between ADC ratio and ISUP grade.

Several other authors have claimed that the ratio, often tumor versus the contralateral normal appearing tissue, is better than the absolute ADC value. Lebovici et al. showed the usefulness of an ADC ratio in differentiating low-grade and high-grade disease (25). Similar results were reported by Boesen et al. and Litjens et al. (8, 27). Interestingly, both absolute ADC values and the ADC ratios differed considerably between these studies. Itatani et al. assessed 58 men who underwent RALP after MRI and used the internal obturator muscle as the ADC reference, finding superior use of the ratio (AUC 0.85 vs. 0.71) (28). Bajgiran et al. concluded that the ADC ratio is a more robust biomarker of PCa aggressiveness (21). Conversely, Rosencrantz et al. found no benefit of using the ADC ratios with urine ADC as the denominator for differentiating benign and malignant tissue in the PZ (17). Woo et al. (20) included 165 men, and DeCobelli 72 men (26), with contralateral prostatic tissue as the reference and found no benefit of the ADC ratio compared to standalone ADC.

Woo et al. pointed out several reasons why the use of the ADC value for internal reference organs may not yield helpful ADC ratios and thereby add, rather than reduce, sources of error in the interpretation (20). For example, they emphasize that the ADC value of the non-tumor PZ can vary according to age, and that the intrinsically organized chaos of the TZ results in a wide range of normal ADC values (29). Moreover, post-biopsy changes can alter the signal intensity of DWI in the prostatic tissue for several weeks. Finally, as hypothesized by DeCobelli, non-tumorous tissue can be affected by nearby non-visible tumor infiltration or by peritumoral fibrosis and inflammation, which all affect the ADC (26). The b-values that were used to estimate the ADC (**Table 2**) varied across MRI systems and sites, and several were inconsistent with PI-RADS recommendations (2). For example, the estimation of ADC based on data acquired at low b-values (<100 s/mm^2^) may introduce a positive bias due to incoherent blood perfusion (30). Furthermore, when the ADC is based on high b-values (>1000 s/mm^2^), the estimation in normal tissue may be negatively biased due to the rectified noise floor (31). These factors may explain why the ratio did not show a better inverse correlation with cancer aggressiveness than standalone ADC. Moreover, in a systematic review of 39 papers with 2457 patients, Surov et al. identified only a moderate correlation between ADC and Gleason score in PCa located in the PZ, and an even worse correlation in the TZ (32).

Harmonizing MRI parameters between centers is important, especially since the ADC values are used for deciding PI-RADS category and hence, affects the clinical decision. In 2007, the Radiological Society of North America organized The Quantitative Imaging Biomarkers Alliance ^®^ (QIBA). QIBA strives for standardization of image acquisition and assesses whether imaging metrics have clinical value (33). Their ongoing work includes evaluation and standardization of DWI in for example MRI Prostate.

In our study, the interrater agreements for different ADC metrics were strong, suggesting that factors other than differences in radiologists’ measurements are the reason for the lack of correlation with pathology. Our results are in line with similar previous studies (19, 23, 34).

Our study has several limitations. First, the study group was small. In addition, the quality of the MRI scans was generally lower than would have been acceptable today. Another limitation is that all included patients had csPCa; therefore, we only obtained data from the more advanced and aggressive tumors. In contrast to previous articles on this topic, no patients with ISUP 1 tumors were subject to prostate resection. This is in line with current clinical treatment guidelines (35). Furthermore, we did not have information on the fraction of Gleason 4 in the ISUP 2 group (Gleason 3 + 4). A lower percentage of Gleason 4 could have put these patients in the group with non-significant cancers. Moreover, the results from pathology were extracted from the original pathology reports, which were produced in a clinical setting by different pathologists with different levels of experience. That is, no study-dedicated pathology examination was performed.

There is potential for improvement, which we will implement in a forthcoming study. Most important is to include the whole range of benign to the most aggressive tumors. This can be achieved by including core biopsies performed using the MR – ultrasound fusion technique. Furthermore, with new digital pathology archives, high precision correlations can be made between the WM RALP specimen and corresponding MR slice. A dedicated revaluation of a specific location in the WM specimen, including tumor subtype and tumor cell growth pattern, can be made.

## Conclusions

In conclusion, our study did not find any correlation between the ADC value and ISUP grade in a multi-scanner setting. We found no benefit of using ADC ratios, so-called normalized ADC values, even with good agreement between the two experienced readers. This contradicts previous single-center studies published research in the field. Therefore, in a clinical situation with different MRI scanner types, measurements of ADC must be used with caution. It also highlights the importance of harmonizing the parameters of the MRI sequences across centers.

## Data availability statement

The raw data supporting the conclusions of this article will be made available by the authors, without undue reservation.

## Ethics statement

The studies involving human participants were reviewed and approved by Lund University (Dnr 2014-886) Swedish ethical review authority (entry no. 2019-03674). Written informed consent for participation was not required for this study in accordance with the national legislation and the institutional requirements.

## Author contributions

JB, ET, DF-S, AB and SZ conceived of the presented idea. JB and ET performed the measurements. DFS retrieved clinical data from patient records. JB, ET, SZ processed the experimental data and performed the analyses. SZ and EB supervised the work. All authors contributed to the writing of the manuscript and approved the submitted version.
