# Supplementary material for: Correlation between ADC, ADC ratio, and Gleason Grade group in prostate cancer patients undergoing radical prostatectomy: Retrospective multicenter study with different MRI scanners
Source: Front Oncol. 2023 Feb 20;13:1079040. doi: 10.3389/fonc.2023.1079040 (PMC9986526; doi:10.3389/fonc.2023.1079040)
Supplement: Supplementary file 1 [file DataSheet_1.docx]

**Supplementary table 1.** Pathological ISUP grade per scanner.

| **Pathological ISUP grade** | **Scanner** | **1** | **2** | **3** | **4** | **5** | **6** | **7** | **8** |
| --- | --- | --- | --- | --- | --- | --- | --- | --- | --- |
|  | **Patients** n = 98 |  |  |  |  |  |  |  |  |
| **1** | 0 | 0 | 0 | 0 | 0 | 0 | 0 | 0 | 0 |
| **2** | 39 | 14 | 5 | 6 | 5 | 3 | 2 | 2 | 2 |
| **3** | 41 | 14 | 5 | 6 | 12 | 4 | 0 | 0 | 0 |
| **4** | 3 | 1 | 0 | 0 | 2 | 0 | 0 | 0 | 0 |
| **5** | 15 | 7 | 2 | 0 | 5 | 0 | 1 | 0 | 0 |
|  | Sum | 36 | 12 | 12 | 24 | 7 | 3 | 2 | 2 |

**Supplementary table 2.** Spearman correlations ADC metrics vs ISUP for 1.5 T and 3 T respectively.

| **Variables** | **Spearmans rho** | **p-value**  **(unadjusted)** |
| --- | --- | --- |
| 1.5 Tesla (n=38) | | |
| ISUP vs. ADC indexlesion | -0.01 | 0.942 |
| ISUP vs. ADC ratio (lesion/kontralateral ref) | 0.19 | 0.254 |
| ISUP vs. ADC ratio (lesion/ PZ ref) | 0.28 | 0.086 |
| ISUP vs. ADC ration (lesion/ urine ref) | 0.31 | 0.060 |
| 3 Tesla (n=60) | | |
| ISUP vs. ADC indexlesion | -0.27 | 0.037 |
| ISUP vs. ADC ratio (lesion/kontralateral ref) | -0.20 | 0.128 |
| ISUP vs. ADC ratio (lesion/ PZ ref) | -0.06 | 0.635 |
| ISUP vs. ADC ration (lesion/ urine ref) | -0.22 | 0.091 |
